# Combining ability of extra‐early biofortified maize inbreds under *Striga* infestation and low soil nitrogen

**DOI:** 10.1002/csc2.20195

**Published:** 2020-06-08

**Authors:** Solomon A. Oyekale, B. Badu‐Apraku, Victor O. Adetimirin

**Affiliations:** ^1^ Pan African Univ. Institute of Life and Earth Sciences (PAULESI) Univ. of Ibadan Ibadan Nigeria; ^2^ Dep. of Crop Production and Soil Science Ladoke Akintola Univ. of Technology P.M.B. 4000 Ogbomoso Oyo Nigeria; ^3^ International Institute of Tropical Agriculture (IITA) P.M.B. 5320 Ibadan Oyo Nigeria; ^4^ Dep. of Agronomy Univ. of Ibadan Ibadan Nigeria

## Abstract

*Striga hermonthica* (Del.) Benth parasitism, low soil N, and nutritional deficiencies of normal‐endosperm maize (*Zea mays* L.) threaten maize yield and exacerbate nutritional problems in sub‐Sahara Africa (SSA). This study was conducted (a) to evaluate genetic variation among extra‐early maturing maize hybrids with provitamin A and quality protein characteristics, (b) to investigate gene action governing the inheritance of *Striga* resistance, grain yield, low N tolerance, and other measured traits under low‐N, high‐N, and *Striga*‐infested environments, and (c) to identify hybrids with high yield and stability across environments. One hundred and fifty hybrids developed using North Carolina Design II were evaluated with six checks under low‐N, high‐N, and *Striga*‐infested environments in Nigeria. Mean squares for hybrids were highly significant (*P* < .01) for grain yield and other traits across environments. Only general combining ability (GCA) for female and/or male mean squares were significant for measured traits under low N. In addition to significant GCA effects for most traits, specific combining ability was significant (*P* < .05) for *Striga* emergence count under *Striga* infestation, and ear height and ears per plant under high N, indicating that additive and nonadditive genetic effects controlled the inheritance of few traits under *Striga* and high N, whereas additive genetic effect governed the inheritance of the traits under low N. Hybrids TZEEIORQ 55 × TZEEIORQ 26, TZEEIORQ 49 × TZEEIORQ 75, and TZEEIORQ 52 × TZEEIORQ 43 were high yielding and stable across environments and have potential for improving nutrition and maize yields in SSA.

**Abbreviations**
AEA: average environment axis
ASI: anthesis–silking interval
ATC: average tester coordinate
DA: days to 50% anthesis
DS: days to 50% silking
EASP: ear aspect
EHT: ear height
EPP: ears per plant
ESP1: *Striga* emergence count at 8 wk after planting
ESP2: *Striga* emergence count at 10 wk after planting
GCA: general combining ability
GCA_f_: general combining ability for female effect
GCA_m_: general combining ability for male effect
GGE: genotype main effect plus genotype x environment interaction
IITA: International Institute of Tropical Agriculture
NCD II: North Carolina Design II
PASP: plant aspect
PC: principal component
PLHT: plant height
PVA‐QPM: provitamin A quality protein maize
QPM: quality protein maize
SCA: specific combining ability
SDR1: *Striga* (host) damage rating at 8 wk after planting
SDR2: *Striga* (host) damage rating at 10 wk after planting
SSA: sub‐Sahara Africa
WAP: weeks after planting
WCA: West and Central Africa.

## 1 INTRODUCTION

Maize (*Zea mays* L.) is an important cereal crop in West and Central Africa (WCA) as well as the dominant staple food and crop in eastern and southern Africa (Edmonds et al., 2009). The average yield of maize in sub‐Sahara Africa (SSA) is 2.4 t ha^−1^ (FAOSTAT, 2017). This is considerably lower than the world average yield of 5.6 t ha^−1^ (FAOSTAT, 2017). Millions of people (especially the poor in rural areas) in SSA subsist on normal‐endosperm maize, deficient in provitamin A (Safawo et al., 2010) and the basic amino acids tryptophan and lysine (Le, Chua, & Le, 2016), resulting in malnutrition and food insecurity in the subregion. The two‐ to more than fourfold projected increase in human population in SSA between 2010 and 2050 and the consequent increase in demand for maize and other major cereals may worsen the food insecurity problem in the region, unless urgent measures are taken (van Ittersum et al., 2016).

The parasitic weed, *Striga*, and low N are among the key stresses that constrain maize yield in SSA (Edmonds et al., 2009; Menkir, Franco, Adepoju, & Bossey, 2012). The biotic stress [*Striga hermonthica* (Del.) Benth] could cause 100% maize yield loss (Fajemisin, 2014), whereas the abiotic stress (low N) may reduce maize yield by close to 50% (Amegbor, Badu‐Apraku, & Annor, 2017). Although there are different methods of *Striga* control (Emechebe et al., 2004; Teka, 2014), resistance and tolerance of host plants is the cheapest and most sustainable strategy to alleviate adverse effects of the weed on maize (Badu‐Apraku et al., 2004). Maize hybrids and varieties that are resistant to *Striga* are the pivot of an integrated *Striga* control strategy (Kim & Adetimirin, 1997). Severe effects of *Striga* on maize are commonly observed in areas with poor soil fertility and low soil N (Sauerborn, Kranz, & Mercer‐Quarshie, 2003). Nitrogen is needed for optimal growth and productivity of maize, but it is traditionally low in tropical soils (Abe, Adetimirin, Menkir, Moose, & Olaniyan, 2013; Betrán, Beck, Banziger, & Edmeades, 2003). Improvement in soil N through application of inorganic fertilizer during maize production in SSA is rarely done, and when carried out, application is at rates considerably lower than recommended rates due to prohibitive prices of fertilizer to resource‐constrained farm families (Amegbor et al., 2017). In effect, maize is usually grown under N stress resulting in low yield of the crop. Although a few varieties and hybrids of maize with *Striga* resistance and tolerance, low N tolerance, and improved provitamin A content have been developed and commercialized in WCA (Badu‐Apraku et al., 2016; Menkir, Maziya‐Dixon, Mengesha, Rocheford, & Alamu, 2017), no maize hybrid with *Striga* resistance and tolerance, tolerance to low N, extra‐earliness, and high provitamin A, lysine, and tryptophan contents is available. Such hybrids, if developed and commercialized, will help to jointly address *Striga* and low N constraints, as well as mitigate the adverse effects of vitamin A deficiency and quality protein malnutrition in WCA.

Combining ability is a crucial test that is carried out in hybrid breeding. It can provide information regarding the genetic effect controlling traits of inbreds (Machida, Derera, Tongoona, & Macrobert, 2010). The test helps in selection of promising lines that could serve as parents of hybrids in maize hybrid programs (Abera, Hussein, Derera, Worku, & Laing, 2016; Hallauer, Carena, & Miranda Filho, 2010). Different reports on gene action controlling *Striga* resistance and tolerance, grain yield, low N tolerance, and other traits of maize lines are available. Additive genetic effect was reported to be more important than nonadditive effect in modulating *Striga* resistance (Amegbor et al., 2017; Gethi & Smith, 2004), whereas nonadditive gene action played greater role in genetic control of *Striga* resistance trait (Akaogu et al., 2019; Kim, 1994). Also, dissimilar reports are available on the genetic effect controlling maize grain yield in low‐N environments. Nonadditive genetic effect regulated grain yield under low N, whereas additive gene action controlled grain yield in high‐N environments (Betrán et al., 2003; Makumbi, Betrán, Banziger, & Ribaut, 2011). In contrast, other authors reported that additive gene action controlled grain yield in low‐N environments, whereas nonadditive gene action regulated yield in high‐N environments (Below, Brandua, Lambert, & Teyker, 1997).

In order to breed extra‐early maize hybrids with tolerance and resistance to many stresses and improved nutritional qualities in SSA, the International Institute of Tropical Agriculture (IITA) began a breeding program in 2011 to develop first‐generation extra‐early inbred lines from the provitamin A *Striga*‐resistant quality protein maize variety 2009 TZEE‐OR2 STR QPM. Seventy‐six extra‐early maturing provitamin A quality protein maize (PVA‐QPM) inbreds were extracted from the population. Although limited information is available on the per se performance of the novel inbreds under *Striga*, low N, and heat and drought stress, no information is available on the performance of the inbreds in hybrid combinations evaluated in low‐N, *Striga*, and high‐N environments. Equally, the genetic effects regulating *Striga* resistance, low N tolerance, grain yield, and other measured agronomic characters of the inbreds are yet to be investigated. Extra‐early‐maturing PVA‐QPM hybrids that combine high yield and stability across low N and *Striga* with good performance under high N are currently not available in WCA. Therefore, this study sought (a) to examine genetic variation among single‐cross extra‐early maize hybrids possessing PVA‐QPM characteristics, (b) to investigate genetic effects for *Striga* resistance, grain yield, low N tolerance, and other characters of extra‐early PVA‐QPM hybrids under low N, high N, and *Striga* conditions, and (c) to identify hybrids that combine high yield and stability across environments.

## 2 MATERIALS AND METHODS

### 2.1 Genetic materials and mating design used

Thirty inbred lines were selected from the lines extracted from the extra‐early‐maturing PVA‐QPM variety 2009 TZEE‐OR2 STR QPM developed by IITA, Nigeria. Selection of the lines was based on their varying responses in *Striga*‐infested and low‐N environments. In addition, kernels with deep orange color and appropriate modification for QPM characteristics (using a light box), indicating the presence of *opaque‐2* recessive alleles (Krivanek, De Groote, Gunaratna, Diallo, & Friesen, 2007), were selected. Pedigree information and reactions of the 30 selected inbreds to *Striga* and low N are shown in Table 1. The 30 inbreds were categorized into six unique sets. Each set comprised five PVA‐QPM inbreds. Of the five lines in each set, one was susceptible to *Striga hermonthica* and/or low N (Table 1). Five inbreds in each group were used as female parents in one set and crossed with another five lines as male parents, in a separate set, in the North Carolina Design II (NCD II) (Comstock & Robinson, 1948). One hundred and fifty extra‐early PVA‐QPM hybrids were generated. These hybrids and six *Striga* and low‐N‐tolerant extra‐early maturing normal endosperm yellow hybrids [TZEEI 79 × TZEEI 9, TZEE‐Y Pop STR C5 × TZEEI 58, TZdEEI 1 × TZdEEI 9, TZEE‐Y Pop STR C5 × TZEEI 82, TZEEI 9 × TZEEI 12, and (TZEEI 82 × TZEEI 79) × TZEEI 95] used as checks were assessed under low N, high N, and artificial *Striga* infestation in Nigeria.

**TABLE 1:** Description of the 30 lines used for the study

Serial no.	Designation	Pedigree	Set	Reaction to *Striga*	Reaction to low N
1	TZEEIORQ^a^ 24	2009 TZEE‐OR2 STR QPM S6 21‐2/6‐1/3‐1/2‐1/2‐1/1	1	Susceptible	Susceptible
2	TZEEIORQ 25	2009 TZEE‐OR2 STR QPM S6 21‐2/6‐1/3‐2/2‐1/2‐1/2	1	Tolerant	Tolerant
3	TZEEIORQ 26	2009 TZEE‐OR2 STR QPM S6 21‐2/6‐1/3‐2/2‐2/2‐2/2	1	Tolerant	Tolerant
4	TZEEIORQ 27	2009 TZEE‐OR2 STR QPM S6 21‐2/6‐2/3‐2/2‐2/2‐2/2	1	Tolerant	Tolerant
5	TZEEIORQ 29	2009 TZEE‐OR2 STR QPM S5 21‐2/6‐3/3‐2/3‐2/3‐2/2	1	Susceptible	Tolerant
6	TZEEIORQ 53	2009 TZEE‐OR2 STR QPM S6 27‐1/5‐2/3‐1/2‐3/3‐1/1	2	Susceptible	Susceptible
7	TZEEIORQ 55	2009 TZEE‐OR2 STR QPM S6 27‐1/5‐3/3‐2/3‐1/2‐1/2	2	Susceptible	Tolerant
8	TZEEIORQ 57	2009 TZEE‐OR2 STR QPM S6 27‐1/5‐3/3‐3/3‐1/1‐1/1	2	Tolerant	Tolerant
9	TZEEIORQ 75	2009 TZEE‐OR2 STR QPM S6 82‐2/2‐2/2‐1/4‐1/3‐1/2	2	Tolerant	Tolerant
10	TZEEIORQ 76	2009 TZEE‐OR2 STR QPM S6 82‐2/2‐2/2‐1/4‐3/3‐2/2	2	Tolerant	Tolerant
11	TZEEIORQ 33	2009 TZEE‐OR2 STR QPM S6 22‐1/3‐1/2‐2/4‐2/2‐1/1	3	Susceptible	Susceptible
12	TZEEIORQ 43	2009 TZEE‐OR2 STR QPM S6 22‐3/3‐2/3‐3/3‐2/3‐2/2	3	Tolerant	Tolerant
13	TZEEIORQ 44	2009 TZEE‐OR2 STR QPM S6 22‐3/3‐3/3‐1/3‐1/3‐1/3	3	Tolerant	Tolerant
14	TZEEIORQ 45	2009 TZEE‐OR2 STR QPM S6 22‐3/3‐3/3‐1/3‐2/3‐2/3	3	Tolerant	Tolerant
15	TZEEIORQ 49	2009 TZEE‐OR2 STR QPM S6 22‐3/3‐3/3‐2/2‐1/1‐1/1	3	Tolerant	Tolerant
16	TZEEIORQ 11	2009 TZEE‐OR2 STR QPM S6 20‐2/2‐3/3‐1/2‐1/2‐1/1	4	Susceptible	Susceptible
17	TZEEIORQ 52	2009 TZEE‐OR2 STR QPM S6 27‐1/5‐2/3‐2/2‐2/3‐1/1	4	Tolerant	Tolerant
18	TZEEIORQ 56	2009 TZEE‐OR2 STR QPM S6 27‐1/5‐3/3‐2/3‐2/2‐2/2	4	Tolerant	Susceptible
19	TZEEIORQ 61	2009 TZEE‐OR2 STR QPM S6 27‐5/5‐1/2‐1/3‐2/3‐1/1	4	Tolerant	Tolerant
20	TZEEIORQ 62	2009 TZEE‐OR2 STR QPM S6 27‐5/5‐1/2‐3/3‐2/3‐1/1	4	Tolerant	Tolerant
21	TZEEIORQ 5	2009 TZEE‐OR2 STR QPM S6 19‐1/2‐2/2‐2/2‐1/2‐1/1	5	Tolerant	Tolerant
22	TZEEIORQ 28	2009 TZEE‐OR2 STR QPM S6 21‐2/6‐3/3‐2/3‐1/3‐1/2	5	Tolerant	Tolerant
23	TZEEIORQ 32	2009 TZEE‐OR2 STR QPM S6 21‐5/6‐1/2‐1/2‐3/3‐1/1	5	Tolerant	Tolerant
24	TZEEIORQ 30	2009 TZEE‐OR2 STR QPM S6 21‐2/6‐3/3‐3/3‐1/3‐1/2	5	Tolerant	Tolerant
25	TZEEIORQ 69	2009 TZEE‐OR2 STR QPM S6 34‐1/1‐3/3‐1/1‐2/4‐1/2	5	Tolerant	Tolerant
26	TZEEIORQ 35	2009 TZEE‐OR2 STR QPM S6 22‐1/3‐1/2‐3/4‐1/2‐1/1	6	Susceptible	Susceptible
27	TZEEIORQ 41	2009 TZEE‐OR2 STR QPM S6 22‐3/3‐1/3‐2/3‐3/4‐1/1	6	Tolerant	Tolerant
28	TZEEIORQ 42	2009 TZEE‐OR2 STR QPM S6 22‐3/3‐2/3‐3/3‐1/3‐1/2	6	Tolerant	Tolerant
29	TZEEIORQ 54	2009 TZEE‐OR2 STR QPM S6 27‐1/5‐3/3‐1/3‐2/2‐1/1	6	Tolerant	Tolerant
30	TZEEIORQ 64	2009 TZEE‐OR2 STR QPM S6 27‐5/5‐2/2‐1/2‐2/2‐2/2	6	Tolerant	Tolerant

a TZEEIORQ, tropical *Zea* extra‐early provitamin A quality protein maize inbred.

### 2.2 Field evaluations and management

The 150 NCD II crosses and the six hybrid checks were studied under low‐ and high‐N conditions at Ile‐Ife (7°28′ N, 4°33′ E; 244 m altitude; 1,350 mm annual rainfall) in 2016. Similarly, the hybrids were examined under low‐ and high‐N environments at Mokwa (9°18′ N, 5°4′ E; 457 m altitude; 1,100 mm annual rainfall) in 2016 and 2017, and under high‐N conditions at Abuja (9°15′ N, 7°20′ E; 300 m altitude; 1,700 mm annual rainfall) in 2017. These translate to three low‐N and four high‐N environments for evaluation of the hybrids and the checks. A 12 × 13 simple lattice design was used in all the evaluations. Continuous maize planting and removal of the stover after every harvest for many years were used to deplete the low‐N fields of N. The total soil P, N, and K were quantified using colorimetric and Kjeldahl digestion method (Bremner & Mulvaney, 1982). At Ile‐Ife, the soil had 0.84 g kg^−1^ of N, 2.05 mg kg^−1^ of P, and 0.36 cmol kg^−1^ of K, whereas the soil at Mokwa had 0.85 g kg^−1^ of N, 6.32 mg kg^−1^ of P, and 0.20 cmol kg^−1^ of K. Based on the results of the soil tests, urea was applied to bring the available N in the high‐N plots to 90 kg N ha^−1^, whereas the low‐N plots were brought up to a total of 30 kg N ha^−1^. Nitrogen application was done at 2 wk after planting (WAP) and 4 WAP in equal‐split doses. Whereas 15 kg N ha^−1^ was applied to the low‐N field at 2 WAP, high‐N plots received 45 kg N ha^−1^ from urea. Also, at 2 WAP, low‐ and high‐N fields received 60 kg P ha^−1^ and 60 kg K ha^−1^ as single superphosphate (P_2_O_5_) and muriate of potash (K_2_O), respectively. At 4 WAP, low‐ and high‐N plots were appropriately top‐dressed with urea to achieve the two N levels used in the study. Plots consisted of single rows, each 4 m in length. Spacing between rows was 0.75 m, and spacing within rows was 0.40 m. At planting, three seeds were sown per hole. Two WAP, seedlings were thinned to two plants per stand in order to achieve 66,666 plants ha^−1^. Weeds were controlled using Primextra and Gramoxone at the rate of 5 L ha^−1^ each of atrazine (2‐chloro‐4‐ethylamino‐6‐isopropylamino‐1, 3, 5‐triazine) and paraquat (*N*,*N′*‐bipyridinium dichloride) as pre‐ and postemergence herbicides, respectively. The chemical weed control was augmented with manual weeding as the need arose.

In addition, the 150 hybrids along with the six checks were assessed under artificial infestation with *Striga hermonthica* at Mokwa and Abuja in 2016 and 2017. Apart from the plot length, which was 3 m under *Striga*, the experimental design, number of replications, and inter‐ and intra‐row spacing were the same as for low‐N and high‐N experiments. Prior to sowing of seeds, artificial *Striga* infestation of the field was carried out according to Kim (1991). Each planting hole was infested with 8.5 g of the sand–*Striga* mixture (containing ∼5,000 germinable *Striga* seeds) prior to planting (Badu‐Apraku et al., 2016). Three seeds were sown per infested hole. At 2 WAP, seedlings were thinned to two plants per stand resulting in 66,666 plants ha^−1^. Under *Striga*, fertilizer (15–15–15 N–P–K) application was carried out 4 WAP at the rate of 30 kg ha^−1^ for N, P_2_O_5_, and K_2_O. Weeds were removed by hand, except *Striga* plants. In all the experiments, fall armyworms (*Spodoptera frugiperda*) were controlled using Ampligo (a.i. 100 g L^−1^ chlorantraniliprole + 50 g L^−1^ lamda‐cyhalothrin) at 300 ml ha^−1^.

### 2.3 Data collection

This was carried out on plot basis under low‐N, high‐N, and *Striga*‐infested environments. Number of days to 50% silking (DS) was the number of days when 50% of the plants in each plot had emerged silks. Number of days to 50% anthesis (DA) was the total number of days that 50% of maize plants in each plot had shed pollen. The difference between DS and DA represented the anthesis–silking interval (ASI). Plant and ear heights were measured as the distance between the base of the plant and the first branch of the tassel, and the distance between the base of the plant and the node carrying the uppermost ear, respectively. Ears per plant (EPP) was estimated as the ratio of the total number of ears harvested per plot to number of plants harvested per plot. Ear aspect (EASP) was assessed on a scale of 1–9, where 1 = large, uniform, clean, and well‐filled ears, and 9 = small, variable, rotten, and partially filled ears. Husk cover was rated on a scale of 1–5, where 1 = husks firmly arranged with ear tip covered, and 5 = husks loosely arranged with ear tip exposed. Under low‐N conditions, stay‐green characteristic (STGR) was rated on a scale of 1–9, where 1 = almost 100% of the leaves were green, and 9 = almost 100% of the leaves were dead. In low‐ and high‐N environments, plant aspect (PASP) was visually rated on a scale of 1–9 based on plant type, where 1 = excellent plant type, and 9 = poor plant type (Badu‐Apraku et al., 2016). Additional data collected under artificial *Striga* infestation included *Striga* (host) damage rating and *Striga* emergence count at 8 and 10 WAP. *Striga* (host) damage was visually scored on a scale of 1–9, where 1 = normal plant growth with no visible symptoms, and 9 = total scorching of all leaves, causing premature death of host plant with no ear formation, whereas *Striga* emergence count was recorded as the number of *Striga* plants that emerged per plot at 8 and 10 WAP (Adetimirin, Aken'Ova, & Kim, 2000). Under low N, grain yield (kg ha^−1^) was estimated from grain weight and grain moisture content and thereafter adjusted to 15%. However, under *Striga* and high N, grain yield (kg ha^−1^) was determined from field weight of cobs, assuming 80% shelling percentage, and subsequently adjusted to 15% moisture content.

### 2.4 Chemical analyses of seed samples of extra‐early PVA‐QPM hybrids for carotenoid, lysine, and tryptophan contents

Seed samples of crosses used for carotenoid, lysine, and tryptophan analyses were obtained through selfing the first and last two plants in each plot of the Design II crosses and checks (Suwarno, Pixley, Palacios‐Rojas, Kaeppler, & Babu, 2014). The samples were obtained from plants under high N at Ile‐Ife and Mokwa in 2016. Self‐pollinated ears per plot, for each location, were separately harvested, dried, and shelled (Azmach, Gedil, Menkir, & Spillane, 2013). The processed seed samples were then stored at 4 °C (for ∼5 mo) in the cold storage equipment of IITA. Subsequently, random samples of 20–30 maize kernels from the top‐yielding and stable PVA‐QPM hybrids along with the best check, obtained from composite grains of the hybrid trials of 2016 at Mokwa and Ile‐Ife, were drawn from the storage. The samples were dispatched to the International Maize and Wheat Improvement Center (CIMMYT) for carotenoid, lysine and tryptophan analyses. The maize kernels of the selected hybrids, at CIMMYT, were frozen at −80 °C and ground to fine powder (0.5 µm). The institute's laboratory protocols for carotenoid analyses—namely, extraction, separation, and quantification by high performance liquid chromatography (HPLC)—were used (Galicia, Nurit, Rosales, & Palacios‐Rojas, 2009). The following carotenoids were determined from each sample: lutein, zeaxanthin, beta‐carotene (all‐*trans*, 9‐*cis*, and 13‐*cis* isomers), and beta‐cryptoxanthin. Total provitamin A content of each hybrid was computed according to Suwarno et al. (2014) as: total provitamin A = 0.5(beta‐cryptoxanthin) + beta‐carotene (all‐*trans* + 9‐*cis* + 13‐*cis* isomers). Quantification of the percentages of tryptophan and lysine in whole grain of the hybrids were carried out according to Nurit, Tiessen, Pixley, and Palacios‐Rojas (2009). Each whole grain sample was ground and defatted using a Kjeldahl apparatus, and an enzyme, papain, was added to hydrolyze the protein. A mixture of glacial acetic acid and H_2_SO_4_ was added to induce a purple color, and the concentration of the color induced was determined with a spectrophotometer at 560 nm. The reading from the spectrophotometer was converted to percentage tryptophan:

Percentageoftryptophan=CorrectedODat560nm×Factor

where corrected optical density (OD) = OD_560‐nm sample_ − OD_560‐nm average of papain blanks_, and

$$\mathrm{Factor}=\frac{\mathrm{Hydrolysate}\;\mathrm{volume}}{\mathrm{Standard}\;\mathrm{curve}\;\mathrm{slope}\;\times \;\mathrm{Sample}\;\mathrm{weight}}$$

### 2.5 Data analysis

Prior to statistical analyses, *Striga* (host) damage at 8 and 10 WAP (SDR1 and SDR2, respectively) and *Striga* emergence counts at 8 and 10 WAP (ESP1 and ESP2, respectively) were log transformed to achieve variance homogeneity (Badu‐Apraku et al., 2010). In this study, each year–location combination was a test environment (Ukalski & Klisz, 2016). Analyses of variance for each environment and across environments were carried out on plot means. In the combined ANOVA across research environments, replicates, environments, and incomplete blocks within replications were considered random factors, whereas genotype was considered a fixed effect (Suwarno et al., 2014).

The ANOVA for the 150 NCD II crosses were pooled over sets for each environment (Hallauer et al., 2010) and across research environments using SAS version 9.4 (SAS Institute, 2012). The hybrid component of the source of variation was divided into variation due to males (sets), females (sets), and female × male (sets) interaction. The *F* tests for male (sets), female (sets), and male × female (sets) mean squares were conducted using male (sets) × environment, female (sets) × environment, and male × female (sets) × environment mean squares, respectively. Mean squares of male (sets) × environment, female (sets) × environment, and male × female (sets) × environment were tested using pooled error mean square (table not shown). The general combining ability (GCA) effects for female and male within sets (GCA_f_ and GCA_m_) and specific combining ability (SCA) for each trait were estimated according to Kearsey and Pooni (1996) as shown below:

$$\mathrm{GC}A_{f}=X_{f}-\mu$$

$$\mathrm{GC}A_{m}=X_{m}-\mu$$

where GCA_f_ and GCA_m _are the GCA effects of female and male parents respectively; *X*_f_ and *X*_m_ are the average performance of a line when it was used as a female and male in crosses, respectively, and µ is the overall mean of crosses in the set. Specific combining ability (SCA) effect for the crosses was estimated as

$$\mathrm{SC}A_{X}=\mathrm{XX}-E_{\left(\mathrm{XX}\right)}$$

meaning

$$\mathrm{SC}A_{X}=\mathrm{XX}-\left(\mathrm{GC}A_{f}+\mathrm{GC}A_{m}+\;\mu \right)$$

where SCA*_X_* is SCA of the cross *X*; XX is the observed mean value of the cross; *E*_(XX)_ is the expected mean value of the cross (on the basis of GCA of parents); and µ is the overall mean of crosses.

Standard errors for testing significance of GCA_m_ and GCA_f_ estimates, for the trait of hybrid, were computed from the mean squares of GCA_m_ × environment and GCA_f_ × environment, respectively, as shown below:

SEforGCAm=MSm×eMSm×ef×e×rf×e×r

SEforGCAf=MSf×eMSf×em×e×rm×e×r

where MS_m × e_ and MS_f × e_ are the mean squares of the interaction between male and environment, as well as female × environment, respectively; *f*, *m*, *r*, and *e* are the number of females, males, replicates, and environments, respectively.

Performance across low‐N, *Striga*, and high‐N environments of extra‐early PVA‐QPM hybrids was examined using a multiple‐trait base index. The base index combined grain yield performance across environments, as well as other important agronomic traits under each environment as seen in Badu‐Apraku et al. (2016) and shown below:

$$\begin{array}{ccc} & & \mathrm{Multiple}-\mathrm{trait}\;\mathrm{base}\;\mathrm{index}=\\ & & \;\left[\begin{array}{c} 2(\mathrm{yield})+\mathrm{EPP}-\mathrm{EASP}-\mathrm{STGR}-\mathrm{PASP}\\ -(\mathrm{SDR}1+\mathrm{SDR}2)-0.5(\mathrm{ESP}1+\mathrm{ESP}2) \end{array}\right] \end{array}$$

where yield and EPP are the grain yield and EPP across environments, respectively; EASP is the ear aspect across environments; STGR is the stay‐green characteristic the in low‐N environment; PASP is the plant aspect across low‐ and high‐N conditions; SDR1 and SDR2 are the *Striga* (host) damage rating at 8 and 10 WAP in the *Striga* environment, respectively; and ESP1 and ESP2 are the number of emerged *Striga* plants at 8 and 10 WAP in the *Striga* environment, respectively.

The means, adjusted for block effect, of each genotype for each measured variable was standardized to reduce the effects of the varying scales used. A positive multiple trait base index value showed tolerance of the genotype to both low N and *Striga*, whereas a negative multiple trait index value indicated susceptibility of the genotype to the stresses (Badu‐Apraku et al., 2015).

Grain yield of the 20 top‐performing and five worst extra‐early PVA‐QPM hybrids (as indicated by the multiple trait base index) along with six checks were subjected to genotype main effect plus genotype × environment interaction (GGE) biplot analysis using genotype x environment analysis with R for Windows (GEA‐R) software (Pacheco et al., 2016). The “mean versus stability” view of the GGE biplot was used to identify hybrids with high yield and stability across *Striga*, low‐N, and high‐N environments. The model used is as shown below:

$$Y_{ij}-Y_{j}=\lambda_{1}\xi_{i1}\eta_{j1}+\lambda_{2}\xi_{i2}\eta_{j2}+\Sigma_{ij}$$

where *Y_ij_* is the average yield of genotype *i* in environment *j*; *Y_j_* is the average yield across all genotypes in environment *j*; λ_1_ and λ_2_ are the singular values for principal components PC1 and PC2, respectively; ξ*_i_*_1_ and ξ*_i_*_2_ are the PC1 and PC2 scores, respectively, for genotype *i*; η*_j_*_1_ and η*_j_*_2_ are the PC1 and PC2 scores, respectively, for environment *j*; and Σ*_ij_* is the residual of the model associated with genotype *i* in environment *j*. The data used for the analysis were not transformed (transform = 0) or standardized (scale = 0) but were environment centered (centering = 2) (Yan, 2001).

## 3 RESULTS

### 3.1 ANOVA for grain yield and other important agronomic traits under low N, high N, *Striga*, and across environments

Results of ANOVA for each environment (low N, high N, and *Striga*) and across environments indicated that hybrid had significant (*P* < .01) mean squares for grain yield and other agronomic traits, except EPP under low N (Table 2) and ear height (EHT) under *Striga* (Table 3). Mean squares of environment and hybrid × environment interaction were significant (*P* < .01) for all traits determined under each environment and across 11 research environments except hybrid × environment effect for yield, EPP, EASP, and PASP under low N (Table 2) and hybrid × environment mean square for EHT under *Striga* (Table 3).

**TABLE 2:** Mean squares obtained from the combined ANOVA for grain yield and other agronomic traits of extra‐early provitamin A quality protein maize hybrids evaluated across low‐ and high‐N environments in 2016 and 2017 at Ile‐Ife, Mokwa, and Abuja in Nigeria

Source of variation^a^	df	Yield	EPP	DS	DA	ASI	EASP	PLHT	EHT	PASP	STGR
		kg ha^−1^	no.	d			1–9	cm		1–9	
Low N											
Env	2	659,741,995^***^	2.34^***^	265.24^***^	329.88^***^	3.64^***^	1.260^***^	426,815.80^***^	106,595.41^***^	0.620^***^	4.390^***^
Sets	5	9,753,105^***^	0.07^**^	78.98^***^	97.71^***^	1.84^***^	0.040^***^	677.07^***^	885.38^***^	0.030^***^	0.040^***^
Env × sets	10	409,789ns^†^	0.03ns	3.54^**^	2.92*	0.36ns	0.004ns	352.00^**^	345.82^***^	0.002ns	0.005ns
Rep (env × sets)	15	303,311ns	0.01ns	0.93ns	1.42ns	0.37ns	0.003ns	166.83ns	129.35ns	0.002ns	0.005ns
Block (env × rep)	72	4,550,732^***^	0.04^***^	3.91^***^	3.44^***^	0.36ns	0.020^***^	520.71^***^	317.00^***^	0.010^***^	0.010^***^
Hybrids	155	1,519,635^***^	0.03ns	10.41^***^	1.43^***^	0.63^*^	0.006^**^	357.24^***^	201.00^***^	0.004^***^	0.008^***^
GCA_m_/sets	24	1,343,211ns	0.03ns	20.40^***^	19.42^***^	0.96ns	0.005ns	638.24^**^	243.74^*^	0.003ns	0.006ns
GCA_f_/sets	24	1,686,096ns	0.02ns	13.54^***^	15.28^***^	0.76^*^	0.005ns	638.03^*^	236.19ns	0.002ns	0.010^**^
SCA/sets	96	971,647ns	0.02ns	1.84ns	1.84ns	0.51ns	0.004ns	158.48ns	109.21ns	0.003ns	0.006ns
Hybrids × env	310	891,820ns	0.03ns	1.73^**^	11.43^**^	0.50^*^	0.004ns	198.34^***^	117.69^**^	0.002ns	0.005^**^
GCA_m_/sets × env	48	1,142,891ns	0.03^*^	1.90ns	1.81^*^	0.69^**^	0.006ns	266.13^***^	119.40ns	0.003^*^	0.006^*^
GCA_f_/sets × env	48	1,063,324ns	0.02ns	2.31^**^	2.10^**^	0.38ns	0.005ns	293.37^***^	139.08^**^	0.002ns	0.005ns
SCA/sets × env	192	785,173ns	0.02ns	1.56ns	1.50ns	0.52*	0.003ns	136.05ns	91.35ns	0.002ns	0.005^**^
Pooled error	360	838,902	0.02	1.36	1.26	0.42	0.005	137.32	87.17	0.002	0.004
High N											
Env	3	980,235,113^***^	8.57^***^	1,380.58^***^	1,279.08^***^	3.85^***^	0.670^***^	385,702.10^***^	185,906.59^***^	0.190^***^	–
Sets	5	15,632,843^***^	0.08^***^	146.98^***^	165.62^***^	3.79^***^	0.050^***^	2,133.51^***^	949.24^***^	0.030^***^	–
Env × sets	15	3,707,493^***^	0.03^*^	4.09^**^	6.92^***^	1.47^***^	0.020^***^	334.91^**^	561.83^***^	0.003ns	–
Rep (env × sets)	20	836,950ns	0.01ns	1.24ns	1.47ns	0.43ns	0.002ns	194.93ns	134.32ns	0.002ns	–
Block (env × rep)	96	2,656,587^***^	0.03^***^	5.56^***^	4.73^***^	0.40ns	0.010^***^	495.48^***^	322.68^***^	0.010^***^	–
Hybrids	155	3,095,245^***^	0.03^***^	17.05^***^	17.34^***^	0.96^***^	0.010^***^	505.04^***^	280.66^***^	0.003^*^	–
GCA_m_/sets	24	3,508,289ns	0.03ns	29.97^***^	26.24^***^	1.25^*^	0.010^**^	683.61^***^	236.83ns	0.005^*^	–
GCA_f_/sets	24	5,600,240^***^	0.05^*^	25.70^***^	22.42^***^	1.59^***^	0.020^**^	808.85^***^	463.53^*^	0.004ns	–
SCA/sets	96	1,267,125ns	0.02^**^	2.62ns	2.34ns	0.43ns	0.010ns	203.18ns	203.34*	0.002ns	–
Hybrids × env	465	1,659,140^***^	0.02^**^	2.44^**^	2.43^***^	0.52^***^	0.010^***^	233.16^**^	189.28^**^	0.004^*^	–
GCA_m_/sets × env	72	2,341,336^***^	0.02^*^	2.54*	2.20^*^	0.75^***^	0.010^***^	205.81ns	159.55ns	0.003ns	–
GCA_f_/sets × env	72	2,029,230^***^	0.03^***^	2.57^*^	2.11ns	0.59^**^	0.010^***^	305.19^***^	243.37^**^	0.004^*^	–
SCA/sets × env	288	1,223,003^**^	0.02ns	2.19ns	2.06^*^	0.36ns	0.010^**^	188.66ns	155.37ns	0.003ns	–
Pooled error	480	960,143	0.02	1.92	1.64	0.37	0.004	159.11	150.47	0.003	–

*Note*. Yield, grain yield; EPP, number of ears per plant; DS, days to 50% silking; DA, days to 50% anthesis; ASI, anthesis–silking interval; EASP, ear aspect, where 1 = large, uniform, clean, and well‐filled ears, and 9 = small, variable, rotten, and partially filled ears; PLHT, plant height; EHT, ear height; PASP, plant aspect, where 1 = excellent plant type, and 9 = poor plant type; STGR, stay‐green characteristic, where 1 = almost 100% of the leaves were green, and 9 = almost 100% of the leaves were dead.

^a^Env, environment; rep, replication; GCA, general combining ability; SCA, specific combining ability; GCA_m_, GCA for male effect; GCA_f_, GCA for female effect.

*Significant at the .05 probability level.

**Significant at the .01 probability level.

***Significant at the .001 probability level.

^†^ns, not significant.

**TABLE 3:** Mean squares obtained from combined ANOVA for grain yield and other agronomic traits of extra‐early provitamin A quality protein maize hybrids evaluated under *Striga* infestation at Mokwa and Abuja in 2 yr and across 11 research environments (low N, high N, and *Striga*‐infested) at Ile‐Ife (2016), Mokwa, and Abuja (2016 and 2017) in Nigeria

Source of variation^a^	df	Yield	EPP	DS	ASI	EASP	PLHT	EHT	HUSK	SDR1	SDR2	ESP1	ESP2
		kg ha^−1^	no.	d		1–9	cm		1–5	1–9		no.	
*Striga* infestation													
Env	3	356,066,215^***^	3.81^***^	389.12^***^	137.45^***^	0.360^***^	14,052.87^***^	50,181.73^***^	0.976^***^	0.79^***^	0.35^***^	660.24^***^	623.22^***^
Sets	5	11,502,129^***^	0.31^***^	172.12^***^	25.56^***^	0.040^***^	228.57ns^†^	180.58ns	0.047^***^	0.18^***^	0.10^***^	5.76^***^	4.93^***^
Env × sets	15	5,136,911^***^	0.16^***^	15.70^***^	9.68^***^	0.060^***^	358.86^**^	144.15ns	0.027^***^	0.04^***^	0.03^***^	1.84^**^	1.86^**^
Rep (env × sets)	20	1,592,176ns	0.04ns	4.19ns	2.21ns	0.004ns	190.72ns	100.68ns	0.005ns	0.01ns	0.00ns	0.91ns	0.75ns
Block (env × rep)	96	4,818,338^***^	0.09^***^	11.60^***^	3.51^***^	0.020^***^	557.25^***^	230.49^***^	0.012^***^	0.03^***^	0.02^***^	2.65^***^	2.69^***^
Hybrids	155	2,082,771^***^	0.07^***^	22.39^***^	4.37^***^	0.010^**^	286.47^**^	136.90ns	0.008^**^	0.02^***^	0.01^***^	1.63^***^	1.81^***^
GCA_m_/sets	24	1,670,351ns	0.09ns	40.53^***^	7.53^*^	0.008ns	481.07^**^	158.20ns	0.009ns	0.02^*^	0.02^*^	2.38^*^	2.72^**^
GCA_f_/sets	24	3,451,170^*^	0.13^**^	31.89^***^	6.98ns	0.010^**^	404.20ns	138.86ns	0.013^*^	0.03^***^	0.02^**^	1.83*	2.71^***^
SCA/sets	96	1,273,554ns	0.04ns	5.28ns	2.35ns	0.005ns	205.13ns	131.14ns	0.004ns	0.01ns	0.01ns	1.23^*^	1.25^*^
Hybrids × env	465	1,490,488^***^	0.05^***^	5.86^***^	3.17^***^	0.010^***^	206.04^**^	124.38ns	0.006^**^	0.01^***^	0.01^***^	1.02^*^	1.01^*^
GCA_m_/sets × env	72	2,169,393^***^	0.07^***^	8.17^***^	3.96^***^	0.020^***^	217.24^*^	131.88ns	0.010^**^	0.01^**^	0.01^**^	1.34^**^	1.23^**^
GCA_f_/sets × env	72	1,507,631^*^	0.05^*^	7.91^***^	5.10^***^	0.010^*^	260.91^**^	138.44ns	0.010^**^	0.01^**^	0.01^**^	0.92ns	0.95ns
SCA/sets × env	288	1,095,362ns	0.04ns	4.55ns	2.36ns	0.006ns	175.76ns	113.28ns	0.004ns	0.01ns	0.005ns	0.93ns	0.92ns
Pooled error	480	1,050,007	0.04	4.11	2.20	0.010	163.65	127.79	0.004	0.01	0.005	0.82	0.82
Across 11 environments													
Env	10	829,772,426^***^	4.94^***^	1,321.59^***^	149.16^***^	0.620^***^	214,309.90^***^	99,728.02^***^	3.762^***^	–	–	–	–
Sets	5	19,955,890^***^	0.16^***^	389.67^***^	20.95^***^	0.070^***^	2,200.07^***^	1,762.54^***^	0.033^***^	–	–	–	–
Env × sets	50	4,352,220^***^	0.09^***^	7.71^***^	4.18^***^	0.030^***^	393.85^***^	313.59^***^	0.019^***^	–	–	–	–
Rep (env × sets)	55	966,040ns	0.02ns	2.23ns	1.06ns	0.005ns	192.27ns	126.34^***^	0.004ns	–	–	–	–
Block (env × rep)	264	3,959,263^***^	0.05^***^	7.31^***^	1.52^***^	0.020^***^	535.67^***^	288.62^***^	0.011^***^	–	–	–	–
Hybrids	155	3,734,831^***^	0.05^***^	47.73^***^	3.35^***^	0.020^***^	383.24^***^	383.24^***^	0.008^*^	–	–	–	–
GCA_m_/sets	24	3,327,566^*^	0.06ns	83.97^***^	5.80^***^	0.020*	1,414.23^***^	419.52^***^	0.007ns	–	–	–	–
GCA_f_/sets	24	6,199,159^***^	0.07^*^	63.89^***^	4.93^***^	0.020^***^	1,419.59^***^	550.56^***^	0.010ns	–	–	–	–
SCA/sets	96	1,510,552^**^	0.03ns	5.20^***^	1.16ns	0.010ns	203.97ns	197.32^**^	0.006ns	–	–	–	–
Hybrids × env	1,550	1,488,203^***^	0.03^***^	3.51^***^	1.53^***^	0.010^***^	160.95^***^	160.95^***^	0.006^***^	–	–	–	–
GCA_m_/sets × env	240	1,921,600^***^	0.04^***^	4.30^***^	1.93^***^	0.010^***^	214.34^**^	139.76ns	0.007^***^	–	–	–	–
GCA_f_/sets × env	240	1,749,478^***^	0.04^***^	4.24^***^	2.18^***^	0.010^***^	297.43^***^	184.83^***^	0.009^***^	–	–	–	–
SCA/sets × env	960	1,055,899ns	0.03ns	2.81ns	1.14ns	0.010ns	175.25ns	132.15ns	0.005ns	–	–	–	–
Pooled error	1,309	960,771	0.02	2.57	1.05	0.010	161.67	133.11	0.004	–	–	–	–

*Note*. Yield, grain yield; EPP, number of ears per plant; DS, days to 50% silking**;** ASI, anthesis–silking interval; EASP, ear aspect, where 1 = large, uniform, clean, and well‐filled ears, and 9 = small, variable, rotten, and partially filled ears; PLHT, plant height; EHT, ear height; HUSK, husk cover, where 1 = husks firmly arranged with ear tip covered, and 5 = husks loosely arranged with ear tip exposed.; SDR1, *Striga* damage rating at 8 WAP, where 1 = normal plant growth with no visible symptoms, and 9 = total scorching of all leaves, causing premature death of host plant with no ear formation; SDR2, *Striga* damage rating at 10 WAP; ESP1, number of emerged *Striga* plant at 8 WAP; ESP2, number of emerged *Striga* plants at 10 WAP.

^a^Env, environment; rep, replication; GCA, general combining ability; SCA, specific combining ability; GCA_m_, GCA for male effect; GCA_f_, GCA for female effect.

*Significant at the .05 probability level.

**Significant at the .01 probability level.

***Significant at the .001 probability level.

^†^ns, not significant.

Under low N, GCA_m_ and GCA_f_ were significant for DS, DA, and plant height (PLHT); only GCA_m_ was significant for EHT, whereas only GCA_f_ was significant for ASI and STGR (Table 2). Specific combining ability was not significant for any characters determined under low N (Table 2). Under high N, five traits (DS, DA, ASI, EASP, and PLHT) had significant mean squares for both GCA_m_ and GCA_f_, whereas SCA was significant for two (EPP and EHT) of the nine traits (Table 2). Significant GCA_m_ mean square alone was observed for PASP, whereas only GCA_f_ was significant for grain yield under high N (Table 2). Under *Striga*, all three combining ability estimates (GCA_m_, GCA_f_, and SCA mean squares) were significant only for ESP1 and ESP2 (Table 3). The GCA_m_ and GCA_f_ were significant for DS, SDR1, and SDR2, and GCA_m_ alone was significant for ASI and PLHT, whereas only GCA_f_ was significant for yield, EPP, EASP, and husk cover under *Striga* infestation (Table 3). None of the three combining ability mean squares were significant for EHT under *Striga* (Table 3). Across 11 environments, GCA_m_ was significant for six of the eight traits, whereas GCA_f_ was significant for seven traits (yield, EPP, DS, ASI, EASP, PLHT, and EHT). The SCA was significant for only three of the eight traits—namely, yield, DS, and EHT (Table 3). Although GCA_f_ × environment and GCA_m_ × environment interaction effects were not significant for grain yield and EASP under low N, they were significant for grain yield and several other traits in each of high N (Table 2), *Striga*, and across environments (Table 3). The SCA × environment effect was significant for two traits (ASI and STGR) under low N, for three traits (grain yield, DA, and EASP) under high N (Table 2), but it was not significant for any of the traits determined under *Striga* (Table 3).

### 3.2 Percentages of hybrid sum of squares attributed to pooled GCA and SCA, as well as maternal effects for grain yield and other agronomic traits of the extra‐early PVA‐QPM under low N, high N, *Striga*, and across environments

Although the percentage contributions of GCA and SCA to hybrid sum of squares revealed greater contribution from SCA to the total variation than GCA for grain yield, EPP, EASP, PASP, and STGR under low N (Table 4), SCA mean squares were not significant for these traits and other traits under the stress. The contribution of SCA sum of squares to hybrid sum of squares was greater than GCA sum of squares for EPP and PASP under high N (Table 4), but SCA mean square was not significant for PASP. Under *Striga*, GCA and SCA made comparable contribution to hybrid sum of squares for grain yield and EASP, but GCA made greater contribution to *Striga*‐related traits (Table 4).

**TABLE 4:** Proportion of sum of squares contributed by general combining ability of the inbreds used as male parents (GCA_m_), GCA of the inbreds used as female parents (GCA_f_), and specific combining ability (SCA) for selected traits of extra‐early provitamin A quality protein maize hybrids evaluated for 2 yr under low N, high N, *Striga*, and across environments at Ile‐Ife, Mokwa, and Abuja in Nigeria

Trait	Environment	GCA_m_	GCA_f_	SCA	GCA_f_/GCA_m_
		%			
Yield, kg ha^−1^	Low N	20	24	56	1.2
	High N	25	39	36	1.6
	*Striga*	16	34	50	2.1
	Across	21	40	39	1.9
No. of ears per plant	Low N	20	14	66	0.7
	High N	15	29	56	1.9
	*Striga*	25	34	41	1.4
	Across	23	28	49	1.2
Ear aspect	Low N	18	18	64	1.0
	High N	23	32	45	1.4
	*Striga*	19	30	51	1.6
	Across	32	24	44	0.8
Plant aspect	Low N	18	16	66	0.9
	High N	23	24	53	1.0
	*Striga*	–	–	–	–
	Across	29	27	44	0.9
Stay‐green characteristic	Low N	15	28	57	1.9
	High N	–	–	–	–
	*Striga*	–	–	–	–
	Across	–	–	–	–
*Striga* (host) damage rating at 8 WAP^a^	Low N	–	–	–	–
	High N	–	–	–	–
	*Striga*	27	40	33	1.5
	Across	–	–	–	–
*Striga* emergence count at 10 WAP	Low N	–	–	–	–
	High N	–	–	–	–
	*Striga*	26	26	48	1.0
	Across	–	–	–	–

^a^WAP, weeks after planting.

The ratio of GCA_f_ sum of squares to GCA_m_ sum of squares for grain yield ranged from 1.2 to 2.1 under low N, high N, *Striga*, and across environments (Table 4). For EPP, GCA_f_ was lower than GCA_m_ only under low N. Under high N, *Striga*, and across environments, the ratio of GCA_f_ to GCA_m_ for EPP ranged from 1.2 to 1.9 (Table 4). Stay‐green characteristic was determined only under low N; for this, the ratio of GCA_f_ to GCA_m_ sum of squares was 1.9. The ratio of GCA_f_ and GCA_m_ for EASP under low N, PASP under high N, and ESP2 was 1.0. The ratio of GCA_f_ to GCA_m_ sum of squares was 1.5 for SDR1.

### 3.3 GCA for male and female effects for grain yield, *Striga* resistance indicator traits, and stay‐green characteristic

Under low‐N conditions, TZEEIORQ 53 and TZEEIORQ 5 of the 30 inbreds evaluated showed significant and positive GCA_f_ effect for grain yield, whereas only TZEEIORQ 64 showed significant positive GCA_m_ effect for this trait (Table 5). Under the high‐N environment, inbreds TZEEIORQ 27, TZEEIORQ 53, TZEEIORQ 56, and TZEEIORQ 5 showed significant and positive GCA_f_ effect for grain yield. Under *Striga* infestation, TZEEIORQ 61 and TZEEIORQ 35 exhibited significant and positive GCA_f_ effect for grain yield (Table 5). Across environments, inbreds TZEEIORQ 27, TZEEIORQ 53, TZEEIORQ 61, TZEEIORQ 5, and TZEEIORQ 35 exhibited significant and positive GCA_f_ effect for grain yield, whereas only TZEEIORQ 64 displayed significant and positive GCA_m_ effect for the trait across environments (Table 5). The GCA_f_ and GCA_m_ effects for STGR were significant and negative for the inbred TZEEIORQ 52. Significant and negative GCA_f_ effect only was detected for STGR in inbreds TZEEIORQ 33 and TZEEIORQ 61. Under *Striga* infestation, 5 of the 30 lines showed significant and negative GCA_m_ or GCA_f_ for SDR2. These were TZEEIORQ 53, TZEEIORQ 33, TZEEIORQ 61, TZEEIORQ 69, and TZEEIORQ 35 (Table 5). Of these, only TZEEIORQ 53, TZEEIORQ 61, and TZEEIORQ 69 had significant and negative GCA_m_ and/or GCA_f_ for ESP2.

**TABLE 5:** Estimates of male and female general combining ability (GCA) effects of 30 extra‐early provitamin A quality protein maize inbred lines from factorial crosses for grain yield (under low N), *Striga* damage, emerged *Striga* plants, and stay‐green characteristic in 2016 and 2017 under low‐N, *Striga*, and high‐N environments at three locations in Nigeria

	Yield under low N		Yield under high N		Yield under *Striga*		Yield across environments		*Striga* damage at 10 WAP		Emerged *Striga* plants at 10 WAP		Stay‐green characteristic	
Line	GCA_m_	GCA_f_	GCA_m_	GCA_f_	GCA_m_	GCA_f_	GCA_m_	GCA_f_	GCA_m_	GCA_f_	GCA_m_	GCA_f_	GCA_m_	GCA_f_
	kg ha^−1^													
TZEEIORQ 24	154.85	−179.41	−61.56	−228.98	−339.85	−230.10	−103.43	−217.23	0.28	0.07	0.27	1.95	0.06	−0.14
TZEEIORQ 25	142.39	72.73	101.09	416.03	176.88	100.84	140.99	209.17	−0.11	−0.16	−0.40	−2.29	−0.07	0.03
TZEEIORQ 26	−3.37	−104.38	467.59	−329.18	54.59	87.15	186.18	−119.05	−0.14	−0.03	−2.37	−0.79	−0.01	0.03
TZEEIORQ 27	−113.70	117.46	−26.63	567.08^a^	−228.91	73.44	−120.58	266.37^a^	0.12	0.10	−1.69	−1.14	0.03	0.01
TZEEIORQ 29	−180.18	93.60	−480.50	−424.95	337.29	−31.33	−103.16	−139.27	−0.15	0.03	4.19^a^	2.27	0.00	0.07
TZEEIORQ 53	−245.18	423.62^a^	−261.04	543.70^a^	−17.38	93.61	−166.76	345.81^a^	−0.42^a^	−0.21	−4.51^a^	−0.6	−0.01	−0.12
TZEEIORQ 55	169.52	236.18	150.50	135.84	140.60	94.55	149.38	148.84	−0.24	−0.05	1.57	1.62	−0.16	−0.04
TZEEIORQ 57	−194.41	−80.65	−317.15	−318.43	−211.80	−117.51	−246.32	−172.12	0.48^a^	−0.06	2.65	−0.97	−0.12	−0.1
TZEEIORQ 75	84.15	−124.56	396.26	−243.51	13.27	5.31	175.63	−120.83	0.05	0.16	0.04	0.17	0.26	−0.07
TZEEIORQ 76	185.92	−454.59^a^	31.43	−117.59	75.31	−75.96	88.06	−201.70	0.13	0.16	0.26	−0.22	0.03	0.32^a^
TZEEIORQ 33	−45.85	−96.89	−237.29	−277.92	−13.68	373.95	−108.92	7.95	0.04	−0.46^a^	−0.38	−2.73	−0.08	−0.28^a^
TZEEIORQ 43	27.55	−101.92	118.68	−41.38	−210.84	−249.95	−24.00	−132.90	0.20	0.37^a^	0.32	−0.52	−0.02	0.18
TZEEIORQ 44	110.19	103.16	−122.46	164.51	−186.39	108.08	−80.04	129.65	−0.03	−0.06	−0.87	−0.52	0.02	0.03
TZEEIORQ 45	−265.89	192.96	474.86	−2.88	74.39	−378.47	123.26	−86.29	−0.14	0.19	0.00	2.23	0.12	0.12
TZEEIORQ 49	173.99	−97.31	−233.78	157.67	336.52	146.40	89.71	81.58	−0.08	−0.04	0.93	1.54	−0.03	−0.05
TZEEIORQ 11	−502.99^a^	−556.78^a^	−618.59^a^	−1019.58^b^	−108.53	−89.35	−388.84^a^	−551.42^b^	−0.03	0.01	5.96^b^	5.89^b^	0.39^a^	0.39^b^
TZEEIORQ 52	306.11	326.63	127.45	348.47	−153.98	−279.46	87.08	111.39	0.31	0.21	−1.24	0.25	−0.34^a^	−0.31^a^
TZEEIORQ 56	205.34	11.51	234.24	518.33^a^	101.43	−274.04	206.81	86.58	−0.07	0.30	−4.45^a^	−1.73	0.03	0.06
TZEEIORQ 61	−52.41	206.39	110.71	200.88	0.77	790.96^b^	34.76	416.46^b^	−0.02	−0.52^a^	1.15	−5.38^b^	−0.1	−0.25^a^
TZEEIORQ 62	155.03	12.25	146.19	−48.10	160.31	−148.10	163.30	−63.01	−0.19	0.00	−1.41	0.97	0.16	0.12
TZEEIORQ 5	−281.05	564.46^a^	254.26	579.83^a^	105.42	153.58	55.31	434.48^b^	0.02	0.09	4.37^a^	3.03^a^	0.09	−0.07
TZEEIORQ 28	163.08	247.95	−181.75	226.44	−104.12	101.53	−62.04	196.40	0.14	−0.11	−0.59	0.13	0.04	−0.07
TZEEIORQ 30	46.75	−221.00	235.99	−101.90	96.29	−363.05	133.80	−222.74	−0.01	0.22	−0.33	0.97	−0.04	−0.16
TZEEIORQ 32	181.77	−350.08	−68.25	−309.50	−105.10	−167.37	−14.27	−308.74^a^	0.07	0.20	0.09	1.36	0.06	0.24
TZEEIORQ 69	−110.54	−241.33	−240.25	−394.87	7.51	275.31	−112.81	−99.40	−0.22	−0.40^a^	−3.54^a^	−5.50^b^	−0.15	0.06
TZEEIORQ 35	−102.24	59.13	−225.59	77.05	125.63	747.58^b^	−62.24	311.18^a^	−0.17	−0.43^a^	−1.76	−1.79	0.06	0.25^a^
TZEEIORQ 41	−170.26	−115.51	−314.54	−349.02	−149.20	−163.58	−211.18	−219.02	−0.31	−0.23	1.40	0.09	0.03	−0.04
TZEEIORQ 42	175.58	27.23	322.58	194.09	264.82	371.25	258.93	215.11	−0.21	−0.33	1.02	2.25	0.08	−0.02
TZEEIORQ 54	−300.96	−126.23	−57.96	−87.69	−450.65	−619.94^b^	−263.81	−289.56^a^	0.63^b^	0.57^a^	−0.20	0.85	−0.21	−0.21
TZEEIORQ 64	397.90^a^	155.39	275.51	165.56	209.40	−335.31	278.30^a^	−17.71	0.07	0.41^a^	−0.46	−1.40	0.03	0.02
**SE±**	195.18	188.27	241.94	225.23	232.88	194.14	132.17	126.11	0.19	0.20	1.73	1.50	0.14	0.11

*Note*. GCA_f_, GCA effects of the inbred used as a female parent; GCA_m_, GCA effects of the inbred used as a male parent.

**^a^**Significantly different from zero at ≥2 SE.

**^b^**Significantly different from zero at ≥3 SE.

### 3.4 Multiple trait base index values for the highest‐ and lowest‐yielding extra‐early PVA‐QPM hybrids along with the best check

Grain yield of extra‐early PVA‐QPM hybrids across research environments (low N, high N, and *Striga*) varied from 2,767 kg ha^−1^ for TZEEIORQ 30 × TZEEIORQ 11 to 4,950 kg ha^−1^ for TZEEIORQ 25 × TZEEIORQ 64 with an average of 3,937 kg ha^−1^ (Table 6). Of the 15 extra‐early PVA‐QPM hybrids with the best base index values along with the best check, Hybrid TZEEIORQ 33 × TZEEIORQ 75 had the best multiple base index value (8.97), whereas the multiple trait base index value was lowest for the best extra‐early yellow check, TZEEI 79 × TZEEI 9 (1.12) (Table 6).

**TABLE 6:** Mean performance for grain yield and other agronomic traits of 15 best and five worst extra‐early maturing provitamin A quality protein maize hybrids with the best check of the 156 hybrids evaluated across 11 (three low N, four *Striga*, and four high N) environments at Ile‐Ife (2016), Abuja, and Mokwa (2016 and 2017) in Nigeria

Hybrid	Grain yield	Ears per plant	Ear aspect^a^	Stay‐green characteristic^b^	Plant aspect^c^	*Striga* damage at 8 WAP^d^	*Striga* damage at 10 WAP^d^	*Striga* emergence count at 8 WAP	*Striga* emergence count at 10 WAP	Multiple‐trait base index
	kg ha^−1^	no.	1–9					no.		
Best										
TZEEIORQ 25 × TZEEIORQ 64	4,950	0.9	3.7	3.5	4.3	4.2	4.4	12	12	8.22
TZEEIORQ 61 × TZEEIORQ 43	4,755	0.9	4.2	2.9	4.7	3.9	4.4	11	11	8.39
TZEEIORQ 49 × TZEEIORQ 75	4,717	0.9	4.3	3.2	5.0	4.0	4.4	10	12	5.96
TZEEIORQ 27 × TZEEIORQ 64	4,700	0.9	3.9	3.4	4.4	4.3	4.9	10	10	6.33
TZEEIORQ 61 × TZEEIORQ 49	4,698	0.9	4.3	2.9	5.0	3.4	3.8	11	11	8.76
TZEEIORQ 44 × TZEEIORQ 55	4,677	0.8	3.9	2.8	4.7	3.8	4.5	9	16	6.77
TZEEIORQ 55 × TZEEIORQ 25	4,675	0.8	4.0	2.7	4.1	4.5	5.0	13	14	6.28
TZEEIORQ 42 × TZEEIORQ 5	4,564	0.8	4.3	3.1	4.9	3.1	4.1	15	18	6.20
TZEEIORQ 53 × TZEEIORQ 26	4,538	0.9	4.2	2.8	4.4	4.2	4.8	10	10	7.31
TZEEIORQ 52 × TZEEIORQ 49	4,442	0.8	4.1	2.4	5.0	3.6	4.5	7	7	8.96
TZEEIORQ 57 × TZEEIORQ 25	4,430	0.8	4.2	2.9	4.3	4.6	4.6	14	12	5.21
TZEEIORQ 52 × TZEEIORQ 43	4,425	0.9	4.2	2.2	4.8	4.5	4.9	14	18	5.46
TZEEIORQ 61 × TZEEIORQ 44	4,375	0.8	4.3	2.8	5.1	3.4	4.1	5	5	8.07
TZEEIORQ 33 × TZEEIORQ 55	4,373	0.8	4.2	2.6	5.1	3.5	4.0	9	10	7.79
TZEEIORQ 33 × TZEEIORQ 75	4,329	0.9	4.1	3.1	4.6	3.7	4.0	8	10	8.97
TZEEI 79 × TZEEI 9 (best check)	3,799	0.9	4.9	3.5	4.8	4.1	4.6	13	14	1.12
Worst										
TZEEIORQ 30 × TZEEIORQ 62	3,501	0.7	5.0	3.4	5.1	5.0	5.5	23	22	−8.64
TZEEIORQ 54 × TZEEIORQ 5	3,327	0.7	5.1	3.3	5.3	5.4	5.9	24	30	−12.24
TZEEIORQ 32 × TZEEIORQ 61	3,326	0.8	4.9	3.7	5.1	5.1	6.0	20	21	−8.58
TZEEIORQ 32 × TZEEIORQ 11	2,952	0.8	5.2	4.8	5.8	5.0	5.6	23	23	−13.10
TZEEIORQ 30 × TZEEIORQ 11	2,767	0.7	5.3	3.5	5.6	5.1	5.7	27	29	−12.47
Mean	3,937	0.8	4.6	3.2	4.9	4.4	5.0	14	16	
Max.	4,950	0.9	5.3	4.8	5.8	5.5	6.0	27	30	
Min.	2,764	0.7	3.7	2.4	4.1	3.1	3.8	5	5	
LSD (0.05)	1,910	0.3	1.6	1.2	1.3	1.7	1.7	16	17	

^a^1 = large, uniform, clean, and well‐filled ears, and 9 = small, variable, rotten, and partially filled ears.

^b^1 = almost 100% of the leaves were green, and 9 = almost 100% of the leaves were dead.

^c^1 = excellent plant type, and 9 = poor plant type.

^d^WAP, weeks after planting. 1 = normal plant growth with no visible symptoms, and 9 = total scorching of all leaves, causing premature death of host plant with no ear formation.

### 3.5 Mean grain yield and stability of extra‐early PVA‐QPM hybrids across 11 (three low N, four *Striga*, and four high N) environments

**FIGURE 1:**
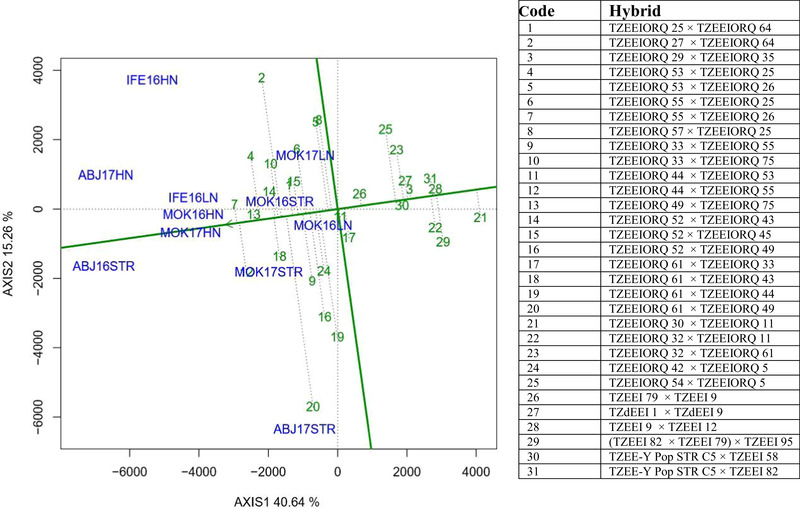
The “mean versus stability” view of the genotype main effect plus genotype x environment interaction (GGE) biplot of grain yield of 20 best and five worst (as indicated by multiple trait base index) extra‐early maturing provitamin A quality protein maize hybrids plus six checks evaluated across 11 (low N, *Striga*‐infested, and high N) environments in Nigeria from 2016–2017: IFE16LN, Ile‐Ife low N 2016; IFE16HN, Ile‐Ife high N 2016; MOK16LN, Mokwa low N 2016; MOK16HN, Mokwa high N 2016; ABJ16STR, Abuja *Striga*‐infested 2016; MOK17LN, Mokwa low N 2017; MOK17HN, Mokwa high N 2017; ABJ17HN, Abuja high N 2017; MOK17STR, Mokwa *Striga*‐infested 2017; ABJ17STR, Abuja *Striga*‐infested 2017

The PC1 of the “mean versus stability” view of the biplot explained 40.6% of the total variation in grain yield of the hybrids, whereas PC2 accounted for 15.3% of the variation in yield across research environments (Figure 1). Hybrids 7 (TZEEIORQ 55 × TZEEIORQ 26), 12 (TZEEIORQ 44 × TZEEIORQ 55), 13 (TZEEIORQ 49 × TZEEIORQ 75), 4 (TZEEIORQ 53 × TZEEIORQ 25), 14 (TZEEIORQ 52 × TZEEIORQ 43), and 18 (TZEEIORQ 61 × TZEEIORQ 43) had the longest projections on the average environment axis (AEA) with above‐mean performance, whereas Hybrids 28 (TZEEIORQ 9 × TZEEIORQ 12) and 21 (TZEEIORQ 30 × TZEEIORQ 11) had the longest projection on AEA with below‐average performance (Figure 1). Nine of the 15 highest‐yielding and most stable PVA‐QPM hybrids selected for provitamin A, tryptophan, and lysine analyses in 2016 had higher provitamin A values than the average provitamin A level (6.06 µg g^−1^) of the hybrids (Table 7). The hybrid with the highest provitamin A content, TZEEIORQ 33 × TZEEIORQ 55 (8.70 µg g^−1^), outperformed the best normal endosperm yellow single‐cross hybrid check, TZEEI 79 × TZEEI 9 (2.86 µg g^−1^), by 67% (Table 7). Also, the level of tryptophan was highest (0.06%) in six hybrids, including the best check, and lowest in two PVA‐QPM hybrids, whereas lysine content was lowest in Hybrid TZEEIORQ 5 × TZEEIORQ 52 and highest in Hybrid TZEEIORQ 64 × TZEEIORQ 30 (Table 7). Hybrid TZEEIORQ 33 × TZEEIORQ 55 combined high levels of provitamin A, tryptophan, and lysine.

**TABLE 7:** Provitamin A, tryptophan, and lysine contents of the composite seeds obtained under high N conditions at Ile‐Ife and Mokwa in 2016 from the 15 highest‐yielding and most stable provitamin A quality protein maize hybrids and the best check across six environments (two environments each of low N, *Striga*, and high N) in Nigeria in 2016

Hybrid	Provitamin A	Tryptophan	Lysine
	µg g**^−1^**	%	
TZEEIORQ 25 × TZEEIORQ 54	5.54	0.04	0.23
TZEEIORQ 26 × TZEEIORQ 35	5.43	0.05	0.26
TZEEIORQ 29 × TZEEIORQ 42	6.32	0.06	0.27
TZEEIORQ 53 × TZEEIORQ 29	6.14	0.05	0.28
TZEEIORQ 55 × TZEEIORQ 26	5.48	0.05	0.28
TZEEIORQ 75 × TZEEIORQ 24	4.45	0.05	0.23
TZEEIORQ 33 × TZEEIORQ 55	8.70	0.06	0.31
TZEEIORQ 52 × TZEEIORQ 45	7.41	0.05	0.24
TZEEIORQ 52 × TZEEIORQ 49	7.61	0.05	0.25
TZEEIORQ 56 × TZEEIORQ 44	7.99	0.04	0.22
TZEEIORQ 61 × TZEEIORQ 43	6.17	0.05	0.21
TZEEIORQ 5 × TZEEIORQ 52	6.80	0.05	0.20
TZEEIORQ 28 × TZEEIORQ 56	4.77	0.06	0.28
TZEEIORQ 42 × TZEEIORQ 30	6.62	0.06	0.27
TZEEIORQ 64 × TZEEIORQ 30	4.72	0.06	0.34
TZEEI 79 × TZEEI 9 (check)	2.86	0.06	0.32
Mean	6.06	0.05	0.26
Max.	8.70	0.06	0.33
Min.	2.86	0.04	0.20
SE	0.37	0.001	0.01

## 4 DISCUSSION

The study addressed the development of maize hybrids with potential to alleviate the nutritional problems under the most important biotic (*Striga hermonthica*) and abiotic (low soil N) constraints to increased maize production and productivity in WCA. Hybrids specifically developed for cultivation in areas infested with *Striga* and with low N are also required to show good performance under optimal growing conditions. This will ensure adaptability to the varied conditions under which maize is cultivated in WCA. This is the first study in WCA aimed at addressing protein quality, provitamin A content, resistance to *Striga*, and tolerance to N stress of extra‐early maize. The highly significant differences detected for grain yield, as well as other agronomic characters, of extra‐early PVA‐QPM hybrids under each environment and across research environments indicated that substantial genetic variability existed among extra‐early PVA‐QPM hybrids studied and that considerable progress could be made from selection for important agronomic traits under the stress environments, as well as nonstress environments. Bhatnagar, Betrán, and Rooney (2004), Langa (2005), and Tilahun et al. (2017) also reported significant differences for grain yield and other traits of QPM lines studied in hybrid combinations, whereas Mushongi (2010) reported significant differences among hybrid maize genotypes evaluated in low‐ and high‐N conditions in Tanzania.

The significant and large environmental variation observed for all the characters determined across environments showed the uniqueness of the environments. This underscores the need for the multi‐environment testing of breeding materials and experimental varieties when pursuing low N tolerance, *Striga* resistance, and good performance in high‐N environments. Similar results were reported by Badu‐Apraku et al. (2013) with normal‐endosperm extra‐early‐maturing yellow inbred lines evaluated under different environments. The lack of significant hybrid × environment interaction mean squares observed for yield, EPP, EASP, and PASP under low N, and EHT under *Striga* environments, indicated that the response patterns of the hybrids were similar for the measured traits in the environments used. The nonsignificant hybrid × environment interaction for these traits can be attributed to the existence of limited variation in the traits among the hybrids under low‐N and *Striga* environments. Nonsignificant hybrid × environment interaction effect for grain yield and EASP under low N, and for EASP under *Striga*‐infested environments, were reported earlier (Ifie, Badu‐Apraku, Gracen, & Danquah, 2015). However, the significant hybrid × environment interaction detected for DS, DA, ASI, PLHT, EHT, and STGR under low N, and all the traits except EHT under *Striga*, indicated that response patterns of the hybrids for these traits were different in the stress environments used in this study. This shows that the influence of environment on the performance of tropical maize depends on the amount of genetic variability present in the traits of the materials used for the study. Menkir, Adetimirin, Yallou, and Gedil (2010) had earlier observed significant hybrid × environment mean squares for grain yield and other *Striga*‐related traits in an evaluation involving hybrid combinations obtained from 10 inbred lines under *Striga* infestation. However, Badu‐Apraku et al. (2013) reported nonsignificant genotype × environment interaction for ESP1 and SDR1 under *Striga* infestation, and STGR under low N. The difference in the reports of the two sets of authors on hybrid × environment interaction for *Striga*‐related traits could be due to the fact that the genetic base of the inbred lines evaluated by Menkir et al. (2010) possessed a broad range of resistance to *Striga hermonthica* compared with the germplasm used by Badu‐Apraku et al. (2013). Although the inbred lines used in this study are of the same extra‐early maturity group as those evaluated by Badu‐Apraku et al. (2013) in hybrid combinations, they interacted differently with the *Striga* environment. This may be because the inbred lines were extracted from a germplasm pool that combines *Striga* resistance with provitamin A and quality protein characteristics.

The significant GCA_m_ and/or GCA_f_ mean squares detected for many characters under each and across research environments showed that additive genetic effect was more important than nonadditive genetic effect in the genetic control of the traits of extra‐early PVA‐QPM studied under low N, high N, *Striga*, and across environments. The mean squares of many traits with higher percentage contribution of SCA sums of squares to the hybrid sum of squares (especially under low N) were not significant, suggesting that nonadditive gene action was less important in regulating those traits. However, the significant SCA mean squares observed for EPP and EHT under high N; ESP1 and ESP2 under *Striga*; and grain yield, DS, and EHT across environments suggested that nonadditive genetic effect was important in the inheritance of the traits under the environments. These results indicated that recurrent selection and hybridization would be useful for genetic improvement of *Striga* tolerance or resistance, low N tolerance, and good performance under high N in a population derived from the extra‐early PVA‐QPM materials studied. This result is in agreement with the report that additive and nonadditive genetic effects played important roles in the inheritance of grain yield and many other traits of early‐maturing provitamin A maize inbreds across *Striga*‐infested and optimal growing conditions in Nigeria (Laban, Badu‐Apraku, & Diakaridia, 2017). Significant GCA_m_ × environment and GCA_f_ × environment effects detected for grain yield and a host of other traits in each environment and across environments indicated that the additive genetic effects for these traits interacted with the environment in their expression. These results justify a multilocation improvement strategy in the development of hybrids with resistance or tolerance to *Striga* and low N, as well as expression of good performance under an optimal N regime.

The NCD II yields two estimates of GCA (GCA_m_ and GCA_f_), a comparison of which provides information on maternal effects. Higher GCA_f_ to GCA_m_ indicated that the cytoplasm had genetic factors that influenced the expression of a trait, in addition to nuclear genes. In this study, the ratio of GCA_f_ effect to GCA_m_ effect was >1 for grain yield and some other measured traits under low N, high N, *Striga*, and across environments, suggesting that maternal effects contributed to the expression of the respective traits of the crosses in each environment and across the environments. The preponderance of GCA_f_ sum of squares to GCA_m_ sum of squares obtained for STGR under low N in this study was a clear indication of a role for maternal effect. Maternal effect was not detected for EASP under low N, PASP under high N, and ESP2. However, the ratio of GCA_f_ sum of squares to GCA_m_ sum of squares was >1 for SDR1. Given the suggestive role of maternal effect on *Striga* damage, a careful choice of female parent is required to exploit favorable cytoplasmic factors for this trait. Maternal effects for grain yield of early‐maturing maize under well‐watered conditions in Nigeria reported by Oyekunle and Badu‐Apraku (2013) are similar to the results obtained under high N in the present study. Therefore, it could be hypothesized that cytoplasmic factors tend to positively influence grain yield under optimal environmental conditions. However, the observation in this study differs from the findings of the earlier authors who reported similar contributions of GCA_m_ and GCA_f_ for grain yield and some traits of early‐maturing normal‐endosperm maize assessed under low‐N, *Striga*, and optimal environmental conditions (Ifie et al., 2015). The differences in the results of this study and that of Ifie et al. (2015) could be attributed to dissimilarities in the genetic materials used and the level of severity of the stresses achieved in the various studies.

The positive and significant GCA_m_ or GCA_f_ effects exhibited for grain yield by inbred lines TZEEIORQ 53, TZEEIORQ 5, and TZEEIORQ 64 under low N and across environments, as well as the significant and positive GCA_f_ effect shown for the trait by TZEEIORQ 61 and TZEEIORQ 35 under *Striga* and across environments, indicated that the lines have potential to contribute favorable alleles for yield in their progenies if used as female or male parent under the stresses and across environments. The significant and negative GCA effect detected in inbred lines TZEEIORQ 52, TZEEIORQ 33, and TZEEIORQ 61 for STGR under low N suggested that progenies of crosses involving these lines are expected to have delayed leaf senescence under N stress. This finding is similar to the reports of Ifie et al. (2015), who obtained negative and significant GCA_f_ effect for STGR in few of the early‐maturing inbred lines studied under low soil N. Desirable inbred lines under *Striga*, in addition to having the capability to reduce host damage and number of emerged *Striga* plants in crosses to other inbred lines, are required to also exhibit high yield in crosses. The significant negative GCA for *Striga* damage score and *Striga* emergence count, and the significant and positive GCA for grain yield of TZEEIORQ 61, indicated that the inbred line has potential for use as a parent when developing *Striga‐*resistant or ‐tolerant PVA‐QPM hybrids. Three of the 15 top‐performing hybrids across environments had inbred line TZEEIORQ 61 as the female parent, suggesting that the line transmitted favorable genes for grain yield, *Striga* tolerance and resistance, and STGR to its progenies. Amegbor et al. (2017) reported significant GCA effects for *Striga* emergence count, *Striga* (host) damage, and STGR in a study of extra‐early maize lines in Nigeria.

High grain yield performance and stability of a genotype is desirable for increased crop productivity (Laban et al., 2017). The hybrids in the present study were ranked along the AEA, with the direction of the arrow indicating a higher mean performance. The long projections of hybrids on either direction away from the biplot origin, on the average tester coordinate (ATC) axis, indicated greater genotype × environment interaction and increased instability. Although Hybrids 12, 4, and 18 were high yielding, they had long projections onto the ATC (i.e., they were relatively unstable). Hybrids 7 (TZEEIORQ 55 × TZEEIORQ 26), 13 (TZEEIORQ 49 × TZEEIORQ 75), and 14 (TZEEIORQ 52 × TZEEIORQ 43) combined long projections on AEA with short projection on ATC, indicating that they combined high grain yield with stability across environments.

The per se provitamin A contents of all the 15 extra‐early PVA‐QPM hybrids selected for provitamin A, lysine, and tryptophan analyses in this study were higher than the provitamin A level of the hybrid with the highest provitamin A content reported by Halilu, Ado, Aba, and Usman (2016) but lower than the provitamin A levels of the best 11 hybrids selected by Suwarno et al. (2014), and the provitamin A concentrations of the extra‐early PVA hybrids TZEEIOR 197 × TZEEIOR 205 (20.1 µg^−1^) and TZEEIOR 202 × TZEEIOR 205 (22.7 µg g^−1^) reported by Badu‐Apraku et al. (2019), indicating the possibility of further improving the provitamin A levels of the extra‐early PVA‐QPM hybrids developed in this study.

## 5 CONCLUSIONS

Substantial genetic variation exists among the extra‐early PVA‐QPM hybrids evaluated in the present study. Under low N, additive genetic effect governed the inheritance of the traits of the inbreds investigated in hybrid combinations, whereas both additive and nonadditive gene action were important in the genetic control of some of the measured traits of the inbreds under high N, *Striga* infestation, and across environments. Maternal effect was observed for grain yield under low N, high N, *Striga*, and across environments, STGR under low N, and EASP and SDR1 under *Striga* infestation. Hybrids TZEEIORQ 55 × TZEEIORQ 26, TZEEIORQ 49 × TZEEIORQ 75, and TZEEIORQ 52 × TZEEIORQ 43 showed high yield and stability across the research environments used in this study. These hybrids have potential for improving nutrition and maize yields under the diverse environmental conditions in WCA.

## AUTHOR CONTRIBUTIONS

S.A.O. and B.B.‐A. conceived, designed, and carried out the experiments; B.B.‐A. provided the genetic materials used for the study; S.A.O. and V.O.A. analyzed the data; S.A.O. drafted the manuscript; S.A.O., V.O.A., and B.B.‐A. interpreted the results. All authors reviewed the manuscript.

## CONFLICT OF INTEREST

The authors declare that there are no conflicts of interest.
